# Enhanced osteogenic commitment of murine mesenchymal stem cells on graphene oxide substrate

**DOI:** 10.1186/s40824-017-0112-8

**Published:** 2018-01-02

**Authors:** Jiyong Kim, Hwan D. Kim, Jungha Park, Eun-seo Lee, Eugene Kim, Seunghun S. Lee, Jin-Kyung Yang, Yoon-Sik Lee, Nathaniel S. Hwang

**Affiliations:** 1 School of Chemical and Biological Engineering, Institute of Chemical Processes, Seoul National University, 1 Gwanak-ro, Gwanak-gu, Seoul, 151-742 Republic of Korea; 2 Interdisciplinary Program in Bioengineering, Seoul National University, Seoul, 152-742 Republic of Korea; 3 N-Bio/BioMAX Institute, Seoul National University, Seoul, 152-742 Republic of Korea

**Keywords:** Graphene, Osteogenesis, Mesenchymal stem cells, Graphene oxide

## Abstract

**Background:**

Tissue engineering is an interdisciplinary field that attempts to restore or regenerate tissues and organs through biomimetic fabrication of scaffolds with specific functionality. In recent years, graphene oxide (GO) is considered as promising biomaterial due to its nontoxicity, high dispersity, and hydrophilic interaction, and these characteristics are key to stimulating the interactions between substrates and cells.

**Method:**

In this study, GO substrates were fabricated via chemically immobilizing GO at 1.0 mg/ml on glass slides. Furthermore, we examined the osteogenic responses of murine mesenchymal-like stem cells, C3H10T1/2 cells, on GO substrates.

**Results:**

C3H10T1/2 cells on GO substrates resulted in increased cell surface area, enhanced cellular adhesions, and instigated osteogenic differentiation. Furthermore, priming of C3H10T1/2 cells with chondrocyte-conditioned medium (CM) could further induce a synergistic effect of osteogenesis on GO substrates.

**Conclusions:**

All of these data suggest that GO substrate along with CM is suitable for upregulating osteogenic responses of mesenchymal stem cells.

## Background

Tissue engineering aims to provide biological living substitutes for damaged tissues or organs from accidents, traumas or diseases [1–4]. As a key component for tissue engineering, stem cells are currently being actively utilized in tissue engineering and regenerative materials field. There have been great improvements for differentiating stem cells, in particular, mesenchymal stem cells, to induce tissue-specific differentiation. However, conventional methods may require several weeks to months of cell culture times for intended cell lineage differentiation [5]. Therefore, effective and facile methods for stem cell differentiation are greatly needed [6]. Recently, modification of biomaterials surface to induce various cellular responses have been investigated [7–10]. These engineered biomaterials can elicit proliferation and differentiation control by selectively interacting with stem cells [11, 12]. Furthermore, it is reported that surface interactions caused by nanotopographies and patterned arrays of coated substrates also have significant effects on differentiation of stem cells [12–14]. As a result, developments of new biomaterial would influence stem cell’s differentiation pathway and its therapeutic application with soluble proteins such as growth factors.

Due to its hydrophilic functional groups and pi-bond electrons on its surface [15], graphene oxide (GO) has been intensively researched for tissue engineering and its applications [16]. Its high electrical conductivity, flexibility, and one-atom-thick layer formation allow GO to be highly capable of interacting with cells, growth factors, and hydrophilic substrate [17, 18]. These properties are attributed to not only functional groups such as carbonyl (CO), carboxyl (-COOH) and hydroxyl (-OH) groups but also pi-bond conjugation by sp2 hybridization. Mesenchymal stem cells cultured on GO-coated surface were reported to exhibit upregulated osteogenic differentiation [19–21]. Mesenchymal stem cells on GO-coated slide showed the increased expression of osteogenic markers, such as osteocalcin (*OCN)*, but down-regulated other markers such as *CD44*, desmin, and *MAP2* [21]. In addition, Nayak et al., has reported that the cells on GO substrate exhibited enhanced adhesion and proliferation [21]. As a result, calcium deposition level and alkaline phosphatase expression were raised. This suggests that GO can be used for osteogenic stimulation of mesenchymal stem cells; it is expected to be an outstanding material as a substrate in the application of not only tissue engineering but also dentistry field for advanced implant and clinical tests.

While it is important to understand the characteristics of GO, it should also consider understanding the background of stem cells and their properties. Stem cells are affected by manipulating material-like mechanics and applying growth factor inducers [22, 23]. Especially during the endochondral ossification process, cartilage is presented by continuous cell division of chondrocytes, which affects the formation of bone tissue. With this phenomenon, *Gerstenfeld* et al. suggested that substances that are secreted from chondrocyte promote osteogenesis [24]. Moreover, our group also demonstrated that C3H10T1/2 cells primed by bovine chondrocyte-conditioned medium (CM) improved osteogenic responses like expressions of osteogenic gene marker like osteocalcin (*OCN*), alkaline phosphatase (*ALP*), collagen type 1 (*COL 1*), and Runt-related transcription factor 2 (*Runx2*). In addition to the previous osteogenic gene markers, these primed C3H10T1/2 cells indicated calcium deposition when it is cultured with osteogenic factors like dexamethasone and ascorbic acid 2-phosphate (A2P) included osteogenic medium. During in vivo test, as a result, bone regeneration rate of critical defect on the mouse calvaria was increased [25].

Based on our previous studies, we hypothesized that GO could bring synergistic effects to C3H10T1/2 cells by priming with CM, complemented with bovine chondrocyte secreted factors. With the characteristics of GO substrate previously stated, chondrocyte secreted factors can have an enhanced influence on stem cell differentiation, leading to up-regulated osteogenesis. These synergistic effects are anticipated to have a significant influence on the osteogenic differentiation experiments in the aspect of improved differentiation induction to the osteogenesis.

## Methods

### Cell priming with conditioned medium

Full-thickness articular cartilage was harvested from the patellofemoral groove and distal femoral condyle of bovine legs as previously described [26]. Collected cartilage tissues were cut into small pieces and incubated with 0.2% type II collagenase (Worthington Biochemical, USA) solution at 37 °C for 16 h. Isolated chondrocytes were filtered with 40 μm mesh and washed with phosphate buffered saline (PBS). To make a conditioned medium, primary bovine chondrocytes were cultured with serum-free medium (15 ml DMEM including 1% Pen Strep) on culture plates (150 mm diameter) for 24 h. The chondrocyte-cultured serum-free medium was then filtered with a 0.2 μm syringe filter and supplemented with 10% FBS (Gibco, USA) to make CM. In order to make a primed cell, C3H10T1/2 cells were cultured with CM for 14 days.

### Graphene oxide (GO) substrate film preparation

Graphite flake and potassium permanganate (KMnO_4_) were purchased from Sigma Aldrich (St. Louis, MO, USA). Sodium nitrate was obtained from Shimakyu’s pure chemical (Osaka, Japan). Sodium hydroxide and sulfuric acid were purchased from Dae-Jung Chemicals (Korea). (GO) was synthesized from graphite flakes by modified Hummers method as previously described [27]. Graphite was oxidized in H_2_SO_4_ and KMnO_4_, then, sonicated for 1.5 h at 12 W in an ice-bath. Subsequently centrifuged and GO was resuspended with H_2_O intensively. Cover glass (1.5 × 1.5 cm) were treated with piranha solution (H_2_SO_4_/H_2_O_2_ (70/30% (*v*/v))) for 1 h, followed by 0.2 N NaOH solution treatment and coated with aqueous GO solution (1.0 mg/ml). GO solution loaded cover glass was kept at 60 °C oven for overnight as loosen lids for covalent bonding between hydroxyl group on the glass surface and epoxy group of GO and slowly evaporated the solution.

### Cell culture and osteogenic differentiation

C3H10T1/2, murine mesenchymal stem cells, were purchased from the Korean Cell Line Bank (KCLB) affiliated to Seoul National University Hospital (SNUH). C3H10T1/2 cells with passage number 26 were plated to the 18 × 18 mm GO/Glass slides at a density of 5 × 10^4^ cells per slide and cultured with Dulbecco’s Modified Eagle Medium (DMEM; GIBCO, USA). The medium was supplemented with 10% fetal bovine serum (FBS; GIBCO, USA) and 1% penicillin/streptomycin (Pen/Strep; GIBCO, USA). For osteogenic differentiation, cells were influenced by osteogenic differentiation substrate, as cultured in DMEM supplemented with 50 mg/ml L-ascorbic acid (Sigma-Aldrich, USA), 1% Pen-Strep, 10% FBS, 100 nM dexamethasone (Sigma-Aldrich, USA), and 10 mM glycerol-2-phosphate (Sigma-Aldrich, USA), for 14 days. Osteogenic differentiation medium was replaced for every day.

### Cell proliferation and viability analysis

Cell proliferation was examined by the Alama Blue Assay Kit (Invitrogen, USA) according to the manufacturer’s instructions. Cells were seeded onto the slides at a density of 5000 cells / cm^2^ and incubated for 4 h with 1:10 Alama blue solution in the medium. After incubation, the Alama Blue solution containing medium is collected and the absorbance is measured using AT / Infinite M200 (TECAN, USA). Live / dead analysis was performed using live/dead viability kits (Invitrogen, USA) after 24 h of cell seeding on each slide. Twenty-four hours after seeding, the cells were incubated for 30 min with a live/dead solution containing calcein-AM and ethidium homodimer-1 (EthD-1). Images were then obtained using an LSM 720 confocal microscope (Zeiss).

### Morphological analysis

After cells were cultured for 4 days with osteogenic differentiation medium (OM), cells were fixed with 10% (v / v) formalin and washed with PBS (3 times). Subsequently, permeabilized using 0.1% Triton X-100 for 30 min. Then, samples were stained with a mixture of 1: 100 Phalloidin (Alexa Fluor 594, life technologies, USA) and 1: 500 vinculin (Abcam, USA) for 1 h. After another 1 h of second antibody treatment, the cells were further stained with DAPI (¢ 6-diamidino-2-phenylindole, Sigma-Aldrich) solution at a ratio of 1: 200 for 15 min. The fluorescence image was obtained with a confocal microscope (ZEISS LSM 720).

### Real time-PCR

RNA samples were obtained from each GO and glass slides (*n* = 4) containing cells with the Trizol method (Trizol®, Life Technology, USA). Total RNA concentration was measured with a NanoDrop spectrometer (ND-2000, NanoDrop Technologies, USA). Each sample was normalized to 1000 ng of total RNA and reverse transcribed into cDNA using the TOPscript ™ Reverse Transcriptase Kit (Enzynomics) according to the manufacturer’s instructions. Real-time PCR was performed with ABI StepOnePlusTM real-time PCR system (Applied Biosystems, USA) using the SYBR Green PCR Master mix. Expressions of genes related with osteogenic differentiation such as *GAPDH, Runx2, ALP,* and *OCN* were analyzed. cDNA samples were loaded and the data were analyzed by the –2^ΔΔCt^ method***.*** PCR primers sequence was as follows***:*** *GAPDH* (forward: 5′-GTA TGA CTC CAC TCA CGG CAA A-3′, reverse: 5’-CTA AGC AGT TGG TGG TGC AG-3′), *RunX2* (forward: 5′-GGA CGA GGC AAG AGT TTC A-3′, reverse: 5′-TGG TGC AGA GTT CAG GCA G-3′), *ALP* (forward: 5′-GAA GTC CGT GGG CAT CGT-3′, reverse: 5’-CAG TGC GGT TCC AGA CAT AG-3′), *OCN* (forward: 5’-AGC AGG AGG GCA ATA AGG-3′, reverse: 5’-CGT AGA TGC GTT TGT AGG C-3′).

### Calcium deposition analysis

To analyze the calcium deposition, Alizarin Red S staining was performed. Cells were incubated with OM for 14 days, then the cells were fixed with 10% (v / v) formalin and washed three times with PBS. To make the ARS solution, 20 mg of Alizarin Red S powder (Sigma-Aldrich, USA) was dissolved in 1 ml of distilled water and the pH was adjusted to 4.1 ~ 4.2 with ammonium hydroxide (NH_4_OH). Fixed cells were stained with ARS solution for 20 min and washed 3 times with distilled water for 5 min. For ARS quantification, 800 μl of 10% (v / v) acetic acid per well was added and incubated at room temperature for 30 min. The cells were collected with a cell scraper, transferred to a 1.5 ml tube, and 500 μl of mineral oil was added. The samples were heated at 85 °C for 10 min and cooled with ice for 5 min. The solution was then centrifuged at 20,000 G for 15 min. After centrifugation, 500 μl of supernatant was collected. Then, 200 μl of 10% ammonium hydroxide was added to the supernatant to complete precipitation. To see the results, Absorbance values ​​were measured with a spectrometer.

### Field emission scanning electron microscopy

Cells seeded on GO / Glass slides were cultured in OM for 4 days, then fixed with 4% paraformaldehyde (Polysciences) for 15 min, subsequently dehydrated with 70–100% ethanol (Daejung Chemical) and treated with Hexamethyldisilazane (HMDS; Daejung Chemical) for 1 h. The sample was visualized with a field emission scanning electron microscope (FE-SEM; JSM-6701F, JEOL) at 20 mA for 100 s after platinum coating.

### Western blotting

Protein samples were collected with M-PER (Mammalian Protein Extraction Reagent) and protein expression was analyzed using 10% (*w*/*v*) SDS (sodium dodecyl sulfate) - polyacrylamide gel electrophoresis (*n* = 3 per group) respectively. Protein was transferred to Immobilon-P membrane (Millipore Corp., USA) and blocked with 5% skim milk in 1× PBS-T (pH 7.5, 0.1% Tween-20). Then, probed with primary antibodies against β-actin (#6276, Abcam, USA) diluted in 1:5000 and vinculin (Abcam, USA) diluted in 1:1000 for overnight in a cold room with gentle agitation. The primary antibody probed proteins were incubated with the secondary antibody, anti-rabbit IgG horseradish-peroxidase conjugated, (#7074, Cell Signaling, USA) in a 1:2000 dilution for 1 h at room temperature. To visualize the protein expressions, the blots were developed by a chemiluminescence detection system (Amersham Bioscience, USA).

### Data analysis

The quantitative data were expressed as the means ± standard deviations. The statistical significances were analyzed by one-way analysis of variance (ANOVA) (*p < 0.05, **p < 0.01).

## Results

### Preparation and characterization of Graphene oxide coated slide

**Fig. 1:**
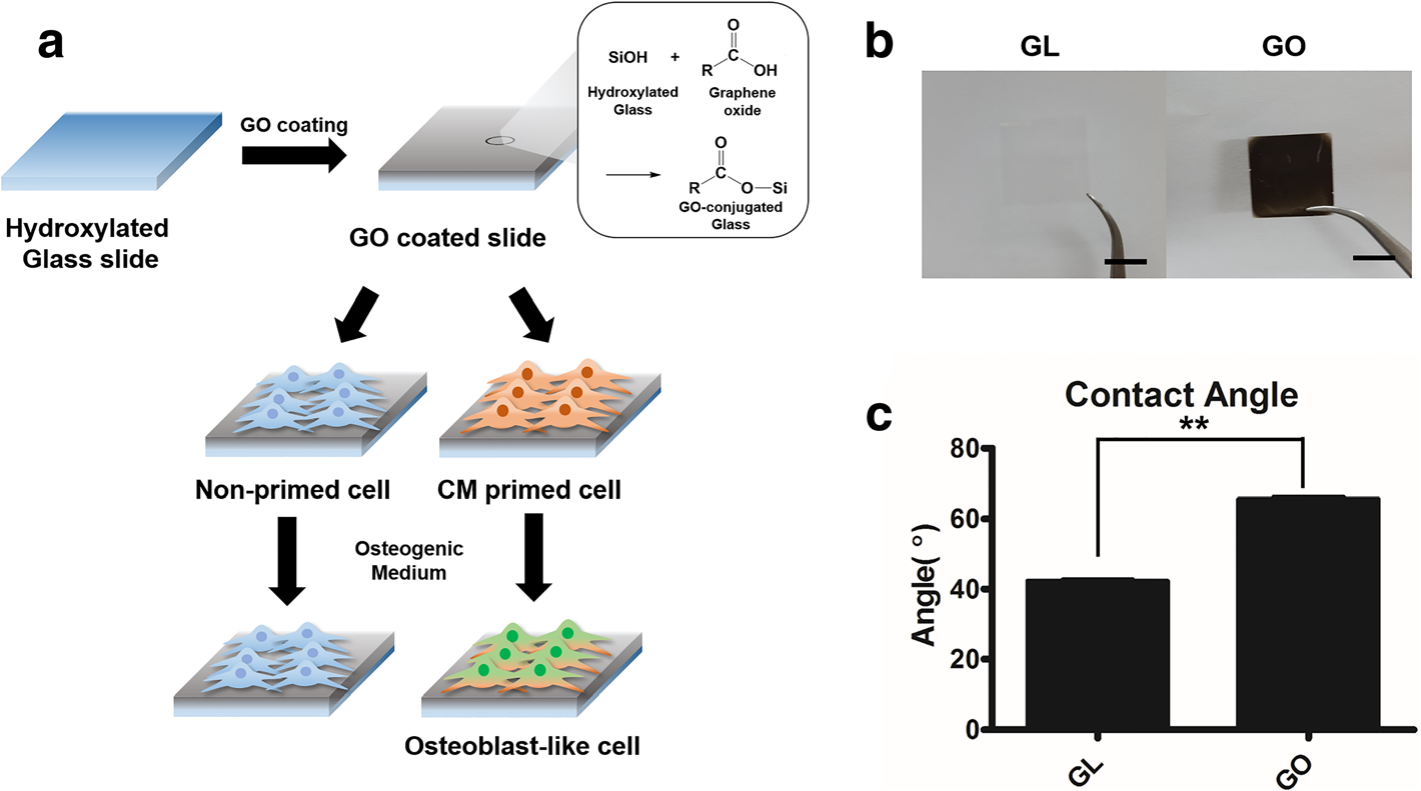
**a** Overall scheme of designing in vitro study with GM/CM primed cell and glass/graphene oxide slide. **b** Image of normal glass slide (Control group) and Graphene oxide substrate coated slide, scale bar = 1 cm. **c** Glass slide and graphene oxide substrate coated slide characterized with water contact angle

Figure 1a represents the overall scheme for graphene oxide coated slide and osteogenic differentiation of C3H10T1/2 cells. Firstly, C3H10T1/2 cells were expanded with either chondrocyte-conditioned medium (CM) or control growth medium (GM) were utilized. Next these cells were cultured on either control glass slide or GO coated slide (Fig. 1a).

GO flakes were made by modified Hummers method [27], and GO flakes were coated on the 18 × 18 mm glass slide. Covalent bonds were formed between hydroxyl group from glass surface treated with piranha solution and epoxy group of GO as pH 10 water slowly evaporated at 60 °C. For the GO coated slides, we observed that the surface of the graphene oxide coated slide formed a dark, thin and opaque layer compared to a glass slide (Fig. 1b). Moreover, the water contact angle was altered due to the GO coating (Fig. 1c). After GO coating, water contact angle was increased about 24°, indicating that the surface area of a GO coated slide was more hydrophobic than a glass slide.

### Cell proliferation, viability, and morphological change

**Fig. 2:**
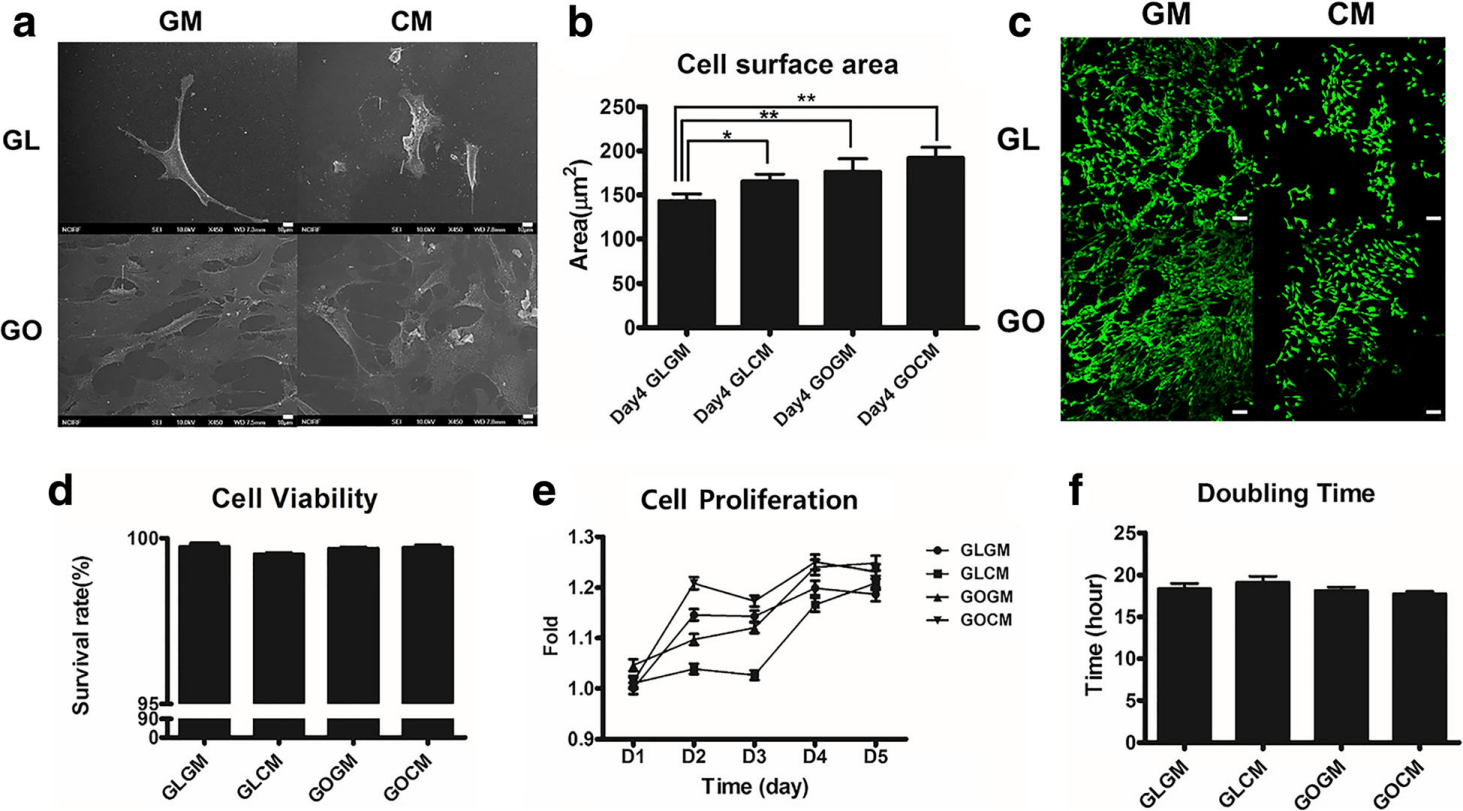
**a** SEM image cells on Glass/GO slide. Cells were cultured for 4 days after seeding, scale bar = 10 μm. **b** Cell surface area of primed/non-primed cell on glass slide. Cells were cultured for 4 days after seeding and measured by image J tools. (*n* = 20, *p < 0.05) (**c**, **d**) Live/dead assay of non-primed/primed C3H10T1/2 cells seeded on Glass/GO slide, scale bar = 100 μm. **e**, **f** Alamar blue assay for each group of C3H10T1/2 cells seeded on Glass/GO slide

To observe the effect of chondrocyte-conditioned medium (CM) priming and GO coated slide, C3H10T1/2 cells were primed for 14 days with CM before seeding. Then, primed and non-primed cells were seeded at a same density on each glass slide or GO coated slide. After 4 days of seeding, cell surface area was altered by CM priming and GO coated slide. When compared to the non-primed cells, priming of C3H10T1/2 cells resulted in 1.18 fold increased in cell size (Fig. 2a and b). The cell size of C3H10T1/2 cells on GO coated slide was 174.3 um^2^ and primed C3H10T1/2 cells on graphene oxide coated slide was 189.2 um^2^_,_ indicating that the both CM expansion increased the cell coverage area.

SEM analysis showed the substrate-dependent adhesion efficiencies (Fig. 2a). On the GO coated slide, there were more cells attached, and the surface area of a single cell was larger than the surface area of a cell on a glass slide. GO substrate is highly capable of interacting with cells due to its hydrophilic property and high adsorption properties for proteins and soluble growth factors. These properties may have resulted in increased cell surface area and adhesion efficiencies.

For the cell viability analysis (Fig. 2c and d), we seeded primed and non-primed C3H10T1/2 cells on either glass slide and GO coated slide. After 1 day of seeding, we measured cellular proliferation and assayed for live/dead. Regardless of the substrate and cell types, all groups showed a similar level of viability (Fig. 2d). All groups showed more than 98% of survival rate indicating that GO or CM did not invoke any significant cytotoxicity to cells.

CM primed cells on glass slide showed decreased proliferation rate compared to the non-primed group while CM primed cells on GO coated slide exhibited improved proliferation rate, as indicated by Alamar Blue assay (Fig. 2e). This suggests that the proliferation of primed cell is enhanced on GO coated slide. The cell doubling time of CM primed cells on GO coated slide showed a statistically significant difference compared with CM primed cells on a glass slide (Fig. 2f). Non-primed cells on glass slide showed a doubling time of 18.4 h, primed cells on the glass slide have a doubling time of 19.2 h, the longest doubling times among the groups. Non-primed cells on GO coated slides have a doubling time of 18.1 h and primed cells on GO coated slides showed a doubling time of 17.6 h, which was the shortest doubling time above all groups. In short, cell doubling times of primed cells were shortened on the GO coated slides for 0.916 times compared to non-primed cells on a glass slide. This result showed that the CM priming had a synergistic effect on the GO coated slides in terms of cellular proliferation. Furthermore, these results demonstrated that a synergistic effect of GO substrate and CM priming could result in morphological change, improved viability, and cellular proliferation.

### Improved focal adhesion of CM primed cells on GO slide

**Fig. 3:**
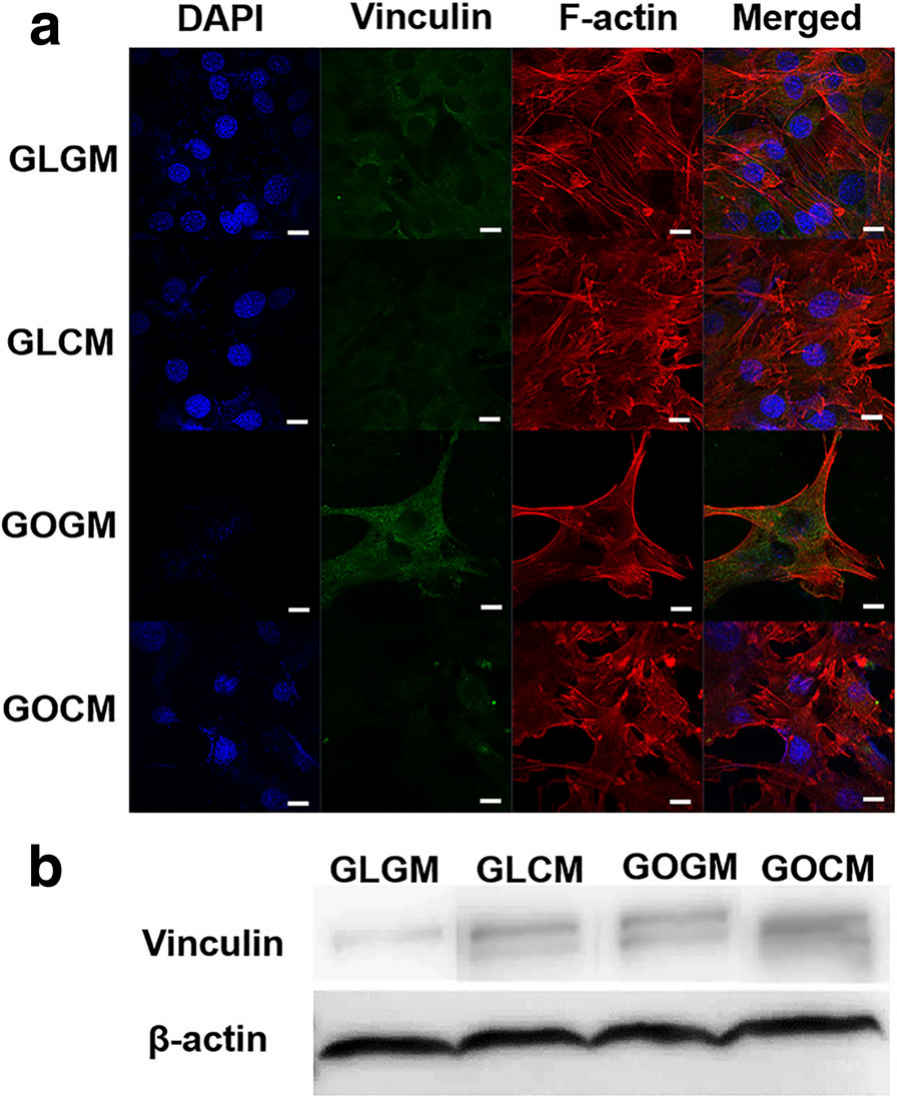
**a** Nucleus, Vinculin and F-actin staining for GM/CM Cell morphology on Glass/GO slide, scale bar = 10 μm. **b** Western blot image to measure Vinculin expressions

Previously, increased cell size coupled with increased focal adhesion kinase (FAK)has been showed to activate cytoskeletal tension via RhoA/Rock signaling pathways, which also enhances osteogenic commitment of stem cells [28]. We cultured primed or non-primed C3H10T1/2 cells on control glass slide of GO slide for 4 days and qualitatively analyzed the F-actin and vinculin by immunostaining (Fig. 3a). F-actin assembly is influenced by cellular tension, cell-ECM adhesion, and cellular surface area. CM primed cells exhibited especially stretched shape to various directions compared to non-primed cells for both on glass slide and GO coated slide (Fig. 3a). Furthermore, with the increased spread of F-actin in the CM primed groups, we showed that CM priming could play a great role to induce the change of cellular morphology and increase cell adhesion. To confirm the correlation between the effect of CM priming and GO slide on focal adhesion, we observed vinculin expression level of each group (Fig. 3b). Similar to previous data, vinculin expression was significantly raised in primed cells on GO coated slides group (GOCM), leading to improving focal adhesion of cells to slide, due to hydrophilicity and pi-bond conjugation of GO substrate. Previous studies showed that contacts with extracellular matrix (ECM) protein induce osteogenic differentiation through a FAK signaling pathway and ERK-dependent pathway [29, 30]. Hence, increased expression of vinculin meant improved focal adhesion and contact with the extracellular matrix protein of cells. With these results, we hypothesized that CM priming and GO coated slide both induced the osteogenic differentiation of C3H10T1/2, along with other synergistic effects when they were used together. To show the cellular morphology and expression of focal adhesion protein increased osteogenic differentiation of C3H10T1/2 cells, we investigated the effect of CM priming and GO coated slide on osteogenic responses by confirming osteogenic gene expressions and bone mineral deposition of each group.

### Osteogenic differentiation of C3H10T1/2 cells

**Fig. 4:**
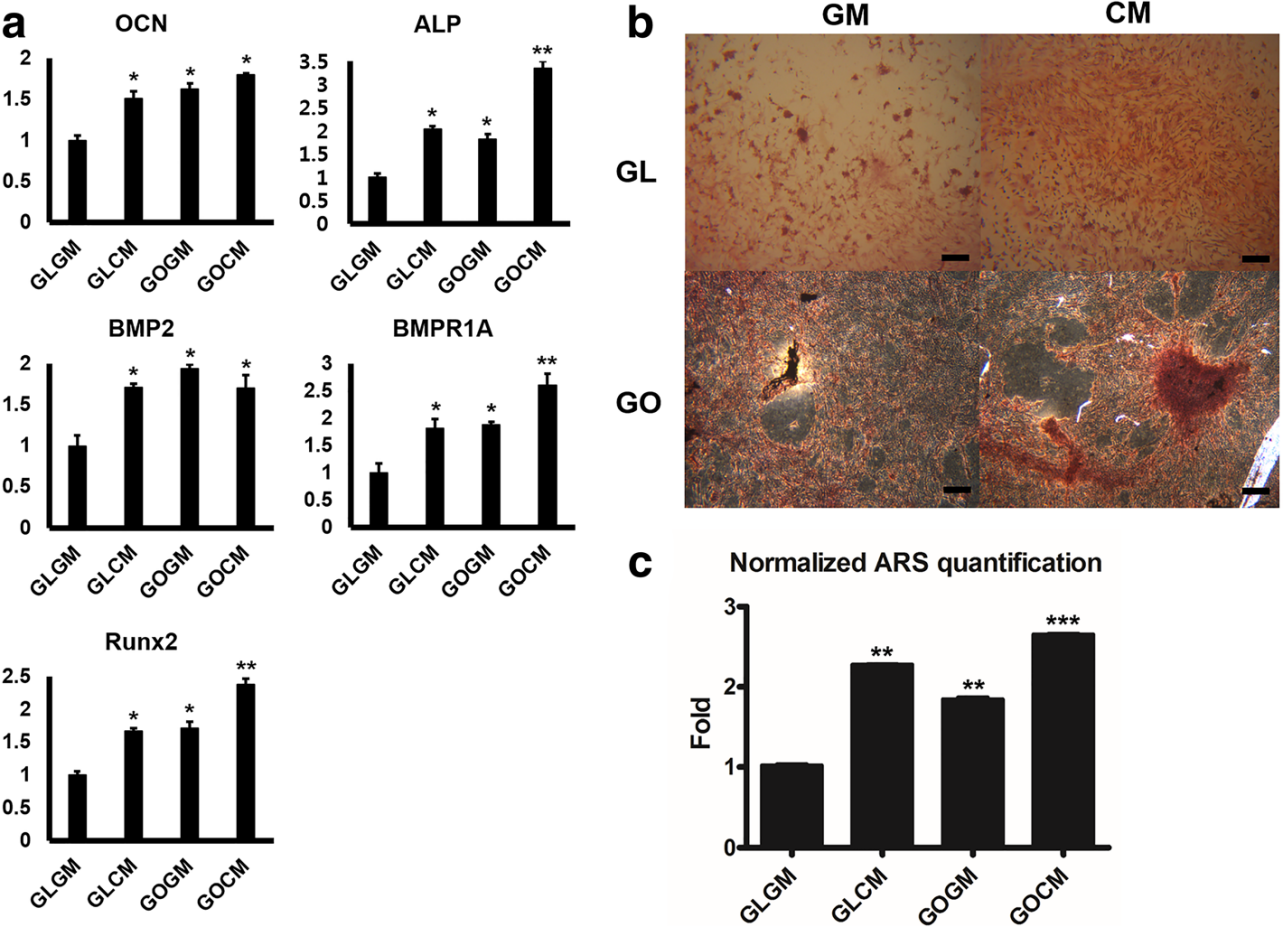
**a** Osteogenic marker expression of the non-primed/primed C3H10T1/2 cells seeded on Glass/GO slide. Real-time PCR was performed after 14 days of osteogenic medium culture on each groups (*p < 0.05, **p < 0.01). **b** Alizarin red staining of non-primed/primed cell on glass slide. Alizarin red staining was performed after 14 days of osteogenic medium culture. Scale bar = 100 μm. **c** ARS staining quantification of non-primed/primed cell on glass/GO slide. Absorbance at 405 nm, normalized by non-primed cell on glass slide (GLGM). The primed cell (CM) has more calcium deposition on it (**p < 0.01, ***p < 0.001)

We cultured CM primed and non-primed C3H10T1/2 cells on each group of slides in the osteogenic medium for 14 days. Osteogenic medium contained ascorbic-2-phosphate, dexamethasone and β-glycerophosphate, which are required for osteogenic differentiation [31, 32]. After culturing cells, osteogenic gene expressions were analyzed by real-time PCR. Compared to those of control group (GLGM), CM primed cells on GO coated slide showed a substantially enhanced osteogenic gene expressions including osteocalcin (OCN, non-collagenous protein found in bone), alkaline phosphatase (ALP, protein promoting osteoblastic activity), runt-related transcription factor 2 (Runx2, early osteogenic gene marker), bone morphogenetic protein 2 (BMP2, essential protein in the development of bone and cartilage), and BMP receptor (BMPR1A) (Fig. 4a). Up-regulation of osteogenic gene expression in CM primed cells on glass slide (GLCM) group and non-primed cells on GO slide (GOGM) was observed, yet primed cells on GO coated slides group (GOCM) were in a higher range of expression.

To determine whether C3H10T1/2 cells differentiated into osteoblast cells, we measured the calcium contents from differentiated cells by Alizarin Red S staining (Fig. 4b and c). On the glass slide, the intensity of ARS stained cells (Red) was more distinguished on CM primed cell. It demonstrated that CM priming could augment the content of calcium deposition. The same phenomenon was observed on the GO slide as well. Furthermore, primed cells on the GO slide presented the greatest calcium deposition rate among all groups. We facilitated ARS quantification to measure the calcium deposition level quantitatively. In agreement with osteogenic gene expression analysis by real-time PCR, cells under the condition of CM or GO slide could deposit more calcium contents than either non-primed cells or cells on glass side. The highest amount of calcium detected in primed cells on GO coated slides group (GOCM) further supported that CM and GO reinforced the synergistic effect in osteogenesis.

## Discussion

Previous studies showed that GO substrate can enhance the osteogenic commitment of stem cells [16]. GO substrate usually functions as cell adhesive platform improving cell adhesion and cell-protein interactions [8, 9]. Furthermore, in a separate study, bovine chondrocyte conditioned medium (CM) priming has been shown to enhance the osteogenic differentiation of mouse or human mesenchymal stem cells [33, 34]. Conditioned medium treating also played a role by improving both chondrogenic and osteogenic responsibility of mesenchymal stem cells through the interaction of cells with the secreted factors from bovine chondrocytes [19, 30].

In this study, we investigated the effect of conditioned medium priming and GO coated slide on osteogenic differentiation of C3H10T1/2 cells, and demonstrated that the synergistic effect of GO coated slide and CM priming could facilitate osteogenesis of C3H10T1/2 (murine mesenchymal stem cells). GO coated surface had increased interaction of extracellular matrix and cells [35]. From our experiment, however, we saw a subtle increase in contact angle. This is due to the carbon components of GO. GO is basically composed of carbon atoms. Usually, long carbon chain shows hydrophobic characters. However, GO coated slide shows hydrophilic features because of its pi bond conjugation and hydrophilic functional groups like -OH and -COOH [15], which may affect the focal adhesion of cell and cell proliferation. Furthermore, the interaction of cells with hydrophilic growth factors like TGF-B groups can be influenced by GO coating. As a result, these properties of GO substrate support that the GO coated surface are highly capable of interacting with cells due its high reactivity to the other proteins and growth factors.

Firstly, we observed that cell surface area from the GO coated slide groups was larger than that from the glass slide groups. Furthermore, the configuration of cell and adhesion rate was different. SEM image showed that there was a significant difference in the cell attachment rate between glass slide group and GO coated slide group, even though both groups had the same amount of days of cell culture with equal cell density. On the GO coated slide, attached cells were more apparent. GO coated slide presented enhanced cell interaction because of its hydrophilic property and these properties also affected the cell morphology such as increased cell surface area and cell adhesion rate.

In addition, CM priming also increased cell surface area. In this experiment, we used CM which contained chondrocyte secreted factors to prime C3H10T1/2 cells. Primed cells on GO slide showed larger surface area than non-primed groups. Among those groups, CM primed cells on GO coated slide showed most stretched shapes to various directions and higher cell adhesion rate, which indicates higher potential and efficiency to be differentiated in direction of osteogenesis [28, 36]. In order to directly observe the cellular response of CM priming and GO substrate, primed C3H10T1/2 cells on GO slide with growth medium (i.e., without osteogenic factors) provide sufficient evidence for cellular commitment. However, our previous study has demonstrated the primed C3H10T1/2 cells cultured with growth medium resulted in stimulated chondrogenic response (i.e., not osteogenic response) [37]. Since our intention in this study was to observe the osteogenic response, we have utilized the osteogenic differentiation medium.

In terms of cell viability and proliferation of each group, primed and non-primed cells on both glass and GO slides acquired more than 98% of survival rate, as shown in Fig. 2d. This demonstrates that GO coating or CM does not have cytotoxicity. As a result, primed cells on glass slide exhibited the longest doubling time among all groups. However, primed cells on GO coated slide showed the shortest doubling time. This result indicates that CM priming facilitates cell proliferation of cells with GO coated slide, not only through conditioned medium’s effect, but also synergistic effect with GO coated slide. As described on Fig. 2a, the cellular morphology of conditioned medium primed cells was especially stretched to various directions compared to non-primed cells. These morphological change on CM primed cells allows the cells to be effectively influenced through contacts with substrate. Moreover, hydrophilic properties of GO can increase the interaction of bovine chondrocyte secreted factors with the cells, which have up-regulating effect on osteogenic differentiation of mesenchymal stem cells [38, 39].

With increased cell surface area, CM primed cell showed stretched F-actin in various directions, and vinculin expression level showed enhancement of focal adhesion for primed cells. Also, increased cell surface area and stretched F-actin on GO slide due to the hydrophilic characters of GO indicates that the effect of focal adhesion protein and extracellular matrix interaction with cells were increased. Therefore, we can speculate that CM primed cells and GO coated slide could have synergistic effects because chondrocyte secreted factors, which had the influence to primed cells, had improved focal adhesion and enhanced the effect of GO substrate on cells. Moreover, it is known that mechanical stimulus on stem cell has a significant effect on its differentiation ability. As the stimulus level increases, the morphology of the cell stretches to various directions and the cell surface area is increases. Those morphological changes shows to have great influence on osteogenic differentiation of the mesenchymal stem cell [28]. In this regard, we demonstrated that the spread of F-Actin, the increased cell surface area of primed cells and, cell growth on GO coated slides enhanced the osteogenic differentiation of cells. In addition, the results of confirming the expression of vinculin, protein related with focal adhesion, through Western blotting had same tendency. The expression level of vinculin was highest at CM primed cells seeded on GO coated slides group.

In this study, furthermore, osteogenic gene marker expressions and ARS staining results supported that the CM priming also to enhance osteogenic responses and calcium deposition. Gene expression level of osteogenic gene markers such as *OCN, ALP, Runx2, BMP2*, and *BMPR1A* were upregulated via CM priming and GO substrate culturing. Simlar to the study by Lee and colleagues, primed cells on GO coated slide showed enhanced osteogenic responses and calcium deposition [40].

## Conclusion

CM priming and GO coated slide affected proliferation, morphological change, and focal adhesions of the C3H10T1/2 cells. CM primed cell with GO slide showed substantially increased osteogenic responses. As a result, CM primed cells seeded on GO slides showed morphological change like increased cell surface and stretched to various direction. In addition, cell proliferation also increased as GO slide was applied. Focal adhesion of mesenchymal stem cells was enhanced as the result is shown at western blot image. This can induce osteogenic differentiation effectively. With the CM priming and GO coated slide, the gene expression level of the osteogenic markers like *OCN, ALP, Runx2, BMP2* and *BMP2R1A* and calcium deposition was upregulated. In short, CM primed cell on GO coated slide showed considerably enhanced osteogenic response compared to any other groups in the experiment. With these results, we could see the synergistic effects of GO and conditioning medium priming. Those synergistic effects of CM priming with GO coating enhanced osteogenic commitment of C3H10T1/2 cells, mesenchymal-like stem cells, and it is possible to utilize these synergistic effects to increase the rate of differentiation of MSCs to osteoblast. Furthermore, CM priming with GO substrate can take part in a solution to excel bone rehabilitation or other therapeutic application such as bone graft therapeutic and, perhaps, tissue engineering as a whole.

## Abbreviations

A2P: Ascorbic 2-phosphate
ALP: Alkaline phosphatase
BMP2: Bone morphogenetic protein 2
BMPR1A: Bone morphogenetic protein receptor 1A
C3H: C3H10T1/2 cells
CM: Chondrocyte-conditioned medium
COL I: Collagen type I
ECM: Extracellular matrix
FAK: Focal adhesion kinase
GL: Glass slide
GLCM: Primed cells on glass slide group
GLGM: Non-primed cells on glass slide group
GM: Growth medium
GO: Graphene oxide
GOCM: Primed cells on graphene oxide slide group
GOGM: Non-primed cells on graphene oxide slide group
OCN: Osteocalcin
RUNX2: Runt-related transcription factor 2
TGF-B: Transforming growth factor-β
